# Novel Imaging-Based Biomarkers for Identifying Carotid Plaque Vulnerability †

**DOI:** 10.3390/biom13081236

**Published:** 2023-08-10

**Authors:** Verónica Fernández-Alvarez, Miriam Linares-Sánchez, Carlos Suárez, Fernando López, Orlando Guntinas-Lichius, Antti A. Mäkitie, Patrick J. Bradley, Alfio Ferlito

**Affiliations:** 1 Department of Vascular and Endovascular Surgery, Hospital Universitario de Cabueñes, 33394 Gijón, Spain; miriamlinares1@gmail.com; 2 Instituto de Investigacion Sanitaria del Principado de Asturias, 33011 Oviedo, Spain; csuareznieto@gmail.com (C.S.); flopez_1981@yahoo.es (F.L.); 3 Department of Otorhinolaryngology, Hospital Universitario Central de Asturias, Instituto Universitario de Oncologia del Principado de Asturias, University of Oviedo, CIBERONC, 33011 Oviedo, Spain; 4 Department of Otorhinolaryngology, Jena University Hospital, 07747 Jena, Germany; orlando.guntinas@med.uni-jena.de; 5 Department of Otorhinolaryngology-Head and Neck Surgery, Helsinki University Hospital, University of Helsinki, P.O. Box 263, 00029 Helsinki, Finland; antti.makitie@helsinki.fi; 6 Research Program in Systems Oncology, Faculty of Medicine, University of Helsinki, 00014 Helsinki, Finland; 7 Division of Ear, Nose and Throat Diseases, Department of Clinical Sciences, Intervention and Technology, Karolinska Institute and Karolinska University Hospital, 17176 Stockholm, Sweden; 8 Department of ORLHNS, Queens Medical Centre Campus, Nottingham University Hospitals, Derby Road, Nottingham NG7 2UH, UK; pjbradley@zoo.co.uk; 9 Coordinator of the International Head and Neck Scientific Group, 35100 Padua, Italy; profalfioferlito@gmail.com

**Keywords:** carotid artery disease, stroke, vulnerable plaque, optical coherence tomography, intravascular ultrasound

## Abstract

Carotid artery disease has traditionally been assessed based on the degree of luminal narrowing. However, this approach, which solely relies on carotid stenosis, is currently being questioned with regard to modern risk stratification approaches. Recent guidelines have introduced the concept of the “vulnerable plaque,” emphasizing specific features such as thin fibrous caps, large lipid cores, intraplaque hemorrhage, plaque rupture, macrophage infiltration, and neovascularization. In this context, imaging-based biomarkers have emerged as valuable tools for identifying higher-risk patients. Non-invasive imaging modalities and intravascular techniques, including ultrasound, computed tomography, magnetic resonance imaging, intravascular ultrasound, optical coherence tomography, and near-infrared spectroscopy, have played pivotal roles in characterizing and detecting unstable carotid plaques. The aim of this review is to provide an overview of the evolving understanding of carotid artery disease and highlight the significance of imaging techniques in assessing plaque vulnerability and informing clinical decision-making.

## 1. Introduction

Traditionally, the clinical assessment of carotid artery stenosis has been based on the degree of luminal narrowing, which has been considered the most reliable predictor of intervention according to international guidelines. The 2017 European Society of Vascular Surgery (ESVS) guidelines [1] and the 2017 European Society of Cardiology (ESC) guidelines [2] were the first to propose new criteria for identifying patients at a higher risk of stroke under best medical treatment (BMT), in whom carotid endarterectomy (CEA) or carotid artery stenting (CAS) might be targeted. Criteria include silent infarction on computed tomography (CT)/magnetic resonance imaging (MRI), 20% stenosis progression, large plaque area or large juxtaluminal black area (JBA) on computerized ultrasound plaque analysis, plaque echolucency, intraplaque hemorrhage (IPH) on MRI, impaired cerebral vascular reserve (CVR), and at least one spontaneous microembolic signal (MES) during 1 h of transcranial Doppler (TCD) monitoring [1,2].

Risk stratification based solely on carotid stenosis has become completely outdated. In fact, in the recently published 2023 ESVS guidelines, patients with only a single risk factor of carotid stenosis greater than 80% are no longer considered to be at high risk of stroke [3]. This stance has been partly influenced by a significant portion of the cohort studies published 20 to 30 years ago but also because of a meta-analysis published by Kamtchum Tatuene and the Asymptomatic Carotid Stenosis and Risk of Stroke Study (ACSRS) group. This demonstrated that increasing stenosis severity was an important predictor for late ipsilateral stroke, but only in the presence of concurrent risk factors [4].

These advancements in the knowledge of the natural progression of atherosclerosis has enabled the identification of distinct characteristics of carotid plaques that are linked to an elevated risk of stroke, consequently introducing the concept of the “vulnerable plaque”, referred to by some as the “unstable plaque” [5,6]. Several structural features have been identified as potential markers of vulnerability, including thin fibrous caps, large lipid cores, IPH, plaque rupture, high macrophage counts, and IPH. Inflammation may also play a role in plaque development and progression. These characteristics reflect an unstable plaque prone to rupture, leading to thrombotic events and subsequent ischemic strokes [7].

Several non-invasive imaging modalities, including US, CT, high-resolution MRI, and nuclear imaging techniques, have been used to identify these plaque characteristics with the aim of achieving an accurate risk stratification and providing guidance for clinical decision-making.

In recent years, advancements and progress in imaging modalities have facilitated the development of intravascular studies, representing a significant breakthrough in the characterization and detection of vulnerable carotid plaques. Given their superior image resolution compared to non-invasive imaging methods, intravascular imaging techniques such as intravascular ultrasound (IVUS), optical coherence tomography (OCT), and near-infrared spectroscopy (NIRS) have emerged as promising modalities for the assessment of culprit lesions in carotid artery disease. However, the translation of these imaging findings into routine clinical practice remains a challenge.

This review aims to provide a comprehensive understanding of the characteristics of unstable carotid plaques, emphasizing the detection of biomarkers derived from novel imaging techniques. In addition, it highlights recent advancements in intravascular carotid imaging techniques and also focuses on the existing clinical evidence supported by non-invasive imaging modalities, elucidating their role in the management of carotid artery disease.

## 2. Biomarkers of Invasive Imaging

### 2.1. Carotid Intravascular Ultrasound (IVUS)

Yock et al. introduced IVUS in 1988 as the pioneering intravascular imaging device specifically designed for coronary applications [8]. Since then, the application of IVUS in carotid artery disease has been explored to assess plaque burden, morphology, and vulnerability, aiding in risk stratification and treatment decision-making.

#### 2.1.1. Fundamental Concepts and Methodological Approaches in IVUS

IVUS utilizes miniature high-frequency transducers (20–45 MHz) placed within an angiographic catheter to obtain real-time, high-resolution images of vascular structures perpendicular to the long axis during pull-back. By analyzing the strength and characteristics of the echoes, IVUS provides valuable information regarding plaque composition and allows for the determination of signs of plaque instability [9].

##### Virtual Histology Intravascular Ultrasound (VH-IVUS)

Although grayscale IVUS can differentiate calcified from non-calcified plaques, it cannot accurately determine non-calcified plaque tissue composition due to post-processing limitations. VH-IVUS utilizes sophisticated radiofrequency analysis of echo signals to generate multiple spectral parameters, which are then converted into color histograms for the analysis of different plaque components [10]. By employing these techniques, VH-IVUS can classify plaques into four phenotypes: fibrous plaque, fibrolipid plaque, necrotic core, and dense calcium, providing a morphological evaluation of the plaque’s evolution [11]. The accuracy of VH-IVUS was validated against histology and has shown a sensitivity, specificity, and predictive accuracy for detecting a necrotic core of 60.1%, 93.0%, and 88.9%, respectively [12].

#### 2.1.2. Features of a Vulnerable Carotid Plaque: Insights from IVUS Imaging

**Figure 1:**
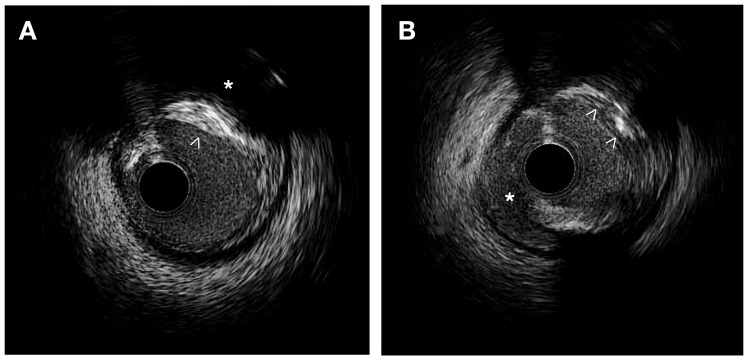
IVUS images. (**A**) Heterogeneous plaque with high echodensity areas (white arrowhead) and back shadowing (*) indicating calcification, as well as lower echodensity zones corresponding to a fibrous plaque. (**B**) Intimal disruption is associated with a dissection (*) and an irregular calcified plaque (white arrowheads).

IVUS provides valuable information for the quantitative assessment of plaque thickness, cross-sectional area, plaque burden, and the remodeling index. It also allows for qualitative assessment, including the identification of the thin fibrous cap and the analysis of plaque composition. This analysis helps differentiate plaque components and assess plaque instability [9,12] (Figure 1).

##### Plaque Composition

Thin cap fibroatheroma (TCFA) is a type of plaque characterized by a plaque burden exceeding 40% and a large necrotic-rich core (>10%), without apparent fibrotic tissue observed in consecutive frames using VH-IVUS [13,14]. In the PROSPECT trial, the presence of a thin fibrous cap evaluated by VH-IVUS demonstrated a significant correlation with the subsequent risk of major adverse cardiac events in patients with acute coronary syndrome [14]. IVUS allows for the identification of lipid-rich plaques, which typically exhibit low echogenicity and appear as a hypoechoic or “dark” region within the vessel wall. In symptomatic patients, IVUS studies using integrated backscatter (IB) analysis have revealed higher percentages of lipid and smaller percentages of fibrous volumes, along with a greater plaque eccentricity, plaque burden, and remodeling index than in asymptomatic patients [15]. High-definition IVUS can identify IPH as an echolucent area with well-defined borders, typically appearing as a crescent-shaped region within the plaque [16]. Using grayscale IVUS, calcium appears as bright, dense, and acoustic shadowing regions and calcified nodules appear as distinct calcifications with an irregular, protruding, and convex luminal surface [17].

##### Plaque Morphology

IVUS allows the quantitative analysis of plaque thickness by means of the measurement of the distance between the luminal surface and the leading edge of the plaque. Comparing cross-sectional areas at different locations within the vessel helps to identify focal stenosis or diffuse disease. It also calculates the percentage of the vessel cross-sectional area occupied by the plaque corresponding to plaque burden. IVUS has confirmed that plaque erosion is characterized by an eccentric plaque with a thick fibrous cap while plaque rupture is observed when a hypoechoic cavity within the plaque is connected to the lumen [18]. Ruptured plaques typically exhibit eccentricity, reduced calcification, increased plaque burden, and positive remodeling, and are often associated with large thrombi [19].

##### Plaque Activity

IVUS-based assessment of inflammatory activity relies on the detection of features such as neovascularization and macrophage infiltration. IVUS can identify neovascularization as microvessels originating from the adventitial side and penetrating into the plaque. It also can calculate the remodeling index by dividing the external elastic membrane area at the site of maximal plaque burden by the average external elastic membrane area in reference segments [20]. The remodeling index reflects the adaptive response of the vessel wall to plaque formation. Numerous clinical studies have demonstrated that pre-interventional positive remodeling, as assessed by IVUS, predicts unfavorable short-term and long-term outcomes following percutaneous coronary intervention [21].

#### 2.1.3. Clinical Practice Perspectives and Emerging Trends in IVUS

##### Diagnostic and Therapeutic Implications

IVUS plays a crucial role in guiding interventional procedures, providing real-time visualization of the vessel and the stented segment, aiding in optimal stent sizing, placement, expansion, and apposition [22]. IVUS enables the longitudinal assessment of plaque progression and regression over time and is relevant in evaluating the effectiveness of therapeutic interventions, such as lipid-lowering therapies or lifestyle modifications. Multiple studies using serial IVUS imaging have demonstrated that statin therapy can slow plaque progression and promote plaque regression in a dose-dependent manner [23,24].

In the prospective multicenter VICTORY registry study, IVUS and IVUS-VH examinations performed during carotid artery interventional therapy were deemed feasible and safe, offering valuable insights into the qualitative and quantitative composition of carotid plaques [25]. Another study conducted by Diethrich et al., entitled The Carotid Artery Plaque Virtual Histology Evaluation (CAPITAL), demonstrated a strong correlation between VH-IVUS plaque characterization and the histological examination of plaques following endarterectomy, particularly for unstable plaque types. They found that the diagnostic accuracy varied with the composition of the plaque (from 99% in TCFA to 72% for calcified atheroma) [26].

Widespread adoption of IVUS to identify risk factors in asymptomatic patients in standard clinical practice is currently facing challenges due to its invasiveness and high cost. Although IVUS demonstrates high sensitivity and specificity in detecting large dense calcified plaques or spot calcifications [27], one of the limitations of IVUS is the limited axial resolution, ranging from 100 to 200 μm. This limitation hinders the identification of thin-fibrous-cap thickness, plaque disruption, macrophage infiltration, and thrombosis within plaques [27]. Combining multiple imaging modalities may help overcome these inherent limitations.

##### Advances in IVUS Technology

Traditional IVUS imaging provides two-dimensional cross-sectional images, limiting the assessment of plaque characteristics in the longitudinal plane. Three-dimensional (3D) IVUS allows for the reconstruction of volumetric images of the vessel and plaque, providing a more comprehensive assessment of plaque burden and morphology. Hybrid imaging systems that combine IVUS with NIRS or OCT catheters allow the simultaneous analysis of both vessel structure and plaque composition and have the potential to overcome the limitations of each technique, thus improving the accuracy of plaque characterization.

The integration of machine learning with artificial intelligence (AI) could be valuable in combining intravascular imaging findings with biomarkers to identify factors associated with plaque instability and progression [28].

### 2.2. Carotid Optical Coherence Tomography (OCT)

Traditionally, OCT has served as a non-invasive imaging diagnostic method that provides valuable insights into the functional blood vessels within the eye and allows for the study of various retinal conditions, such as macular telangiectasia, impaired perfusion, microaneurysms, capillary remodeling, certain types of intraretinal fluid, and neovascularization [29]. In 1991, Huang et al. introduced OCT as an intravascular imaging technique to overcome the limitations of angiography in visualizing the coronary artery [30]. Since 2010, the use of OCT has significantly expanded, particularly in the field of interventional cardiology, and by extension, in carotid artery disease.

#### 2.2.1. Fundamental Concepts and Methodological Approaches in OCT

OCT is an invasive microscopic imaging technology that utilizes reflected near-infrared light and interferometry to generate high-resolution images of the tissue microstructure of the carotid artery with exceptional clarity [31,32].

The OCT system employs rapid scanning of the catheter to acquire multiple cross-sectional images, known as B-scans, along the length of the artery. The catheter is slowly withdrawn, and the scanning process is repeated at different pullback speeds to capture a three-dimensional representation of the structure of the vessel wall and assess plaque morphology, composition, and vulnerability [33] as well as stent positioning in carotid arteries [34].

#### 2.2.2. Features of Vulnerable Carotid Plaque: Insights from OCT Imaging

**Figure 2:**
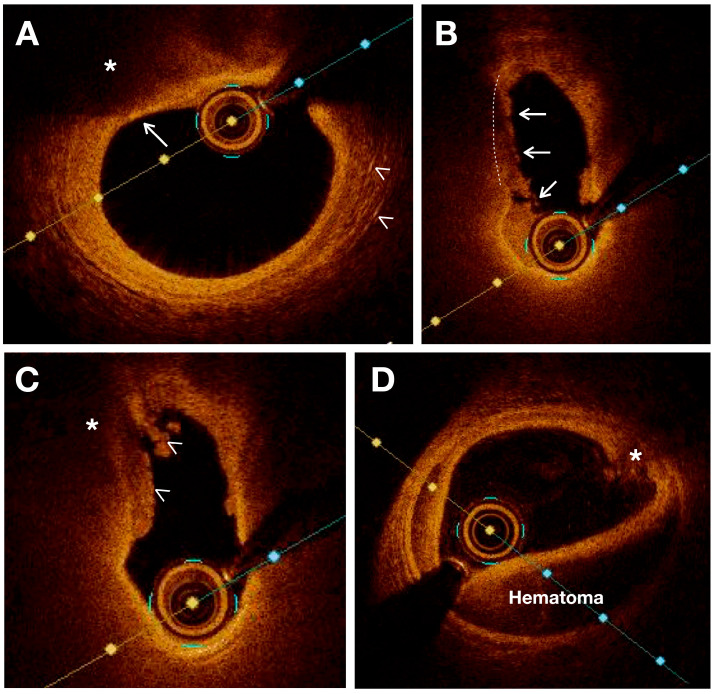
OCT images showing various characteristics of vulnerable plaques. (**A**) Thin fibrous plaque (white arrow) overlying a large lipid pool or necrotic core (*). White arrowheads indicate the presence of cholesterol crystals. (**B**) Plaque rupture (white lines) is sealed with thrombus (white arrows). (**C**) Intraluminal thrombus (white arrowheads) covers a disrupted plaque with necrotic core (*) (**D**) Intramural hematoma and intimal disruption (*) are observed.

In terms of vulnerable plaques, OCT provides visualization and assessment of various characteristics such as cap thickness (with thin caps defined as those ≤65 μm), lipid core detection, calcification, cholesterol crystallization, IPH, plaque erosion, plaque rupture or thrombi, and neovascularization. Moreover, OCT allows for the observation and quantification of inflammation within unstable plaques by measuring macrophage infiltration in the fibrous cap and subintimal lipid accumulation [35]. Previous studies have validated the use of OCT for assessing various characteristics of atherosclerotic plaque using histologic controls. [31,35,36] (Figure 2).

##### Plaque Composition

Lipid cores appear as low-intensity regions with distinct borders on OCT images. Necrotic cores appear as a low-signal area in OCT imaging with an indistinct border, an absence of backscattering signal, and a rapid signal drop-off. OCT also demonstrates high sensitivity and specificity in detecting lipid-rich plaques, as verified by autopsy specimens (90–94% and 90–92%, respectively) [37]. OCT can also visualize fresh and organized intraplaque hemorrhage as high-intensity regions within the plaque due to the presence of red blood cells. Unlike IVUS, OCT can penetrate plaque calcification and provide detailed information regarding its thickness, area, and volume [38]. As a result, the calcified nodules are distinctly delineated from other components of the plaque with a very high sensitivity (96%) and specificity (97%) [36,39].

##### Plaque Morphology

OCT provides a highly detailed visualization of intimal thickening, where the intima layer exhibits a strong backscattering signal at its internal boundary, gradually decreasing in intensity towards the outer layers [40]. Measurements of cap thickness by OCT have been associated with the prevalence of plaque rupture [22]. The hallmark of plaque erosion is represented by a thrombus covering a non-disrupted fibrous cap. On the other hand, plaque ulceration is observed in OCT imaging as an intra-plaque cavity while plaque erosion can occur without involvement of the lesion’s lipid core [39].

##### Plaque Activity

In OCT imaging, inflammation is characterized by a highly intense speckle signal observed in regions adjacent to the fibroatheroma, varying in size. It is crucial to differentiate these areas from cholesterol crystals, elastic lamina, or calcium deposits [41]. It should be noted that OCT indications of inflammation can only be interpreted when a fibrous plaque is present, as there is currently no confirmed information regarding the significance of images suggesting macrophage accumulation in other regions of the vascular wall [41].

#### 2.2.3. Clinical Practice Perspectives and Emerging Trends in OCT

##### Diagnostic and Therapeutic Implications

The clinical applications of OCT in carotid stenosis encompass both diagnostic and therapeutic purposes. The use of OCT in characterizing vulnerable carotid plaques has significant clinical implications for risk stratification and patient management. By providing detailed information on plaque morphology, fibrous cap thickness, lipid-rich regions, and surface features, OCT can help to identify high-risk plaques that are prone to rupture and subsequent ischemic events. This information can assist in determining the optimal treatment strategy for individual patients and the need for invasive interventions.

OCT plays a crucial role in guiding therapeutic interventions for carotid artery disease. It provides real-time feedback during interventions, revealing features that were not visualized using other imaging modalities like CT and MR angiography, such as free intraluminal thrombus, dissection, TCFA with an underlying ulcerative plaque and filling defects inside or adjacent to stents, or an undersized stent [42]. In view of this, several studies have described the use of OCT imaging to guide appropriate endovascular therapy, allowing for accurate stent placement and the detection of plaque prolapse and stent strut malposition during CAS [42,43,44,45,46].

However, the prognostic value of plaque morphology and composition in terms of stroke risk has not been established in large prospective studies. Therefore, it remains challenging to set up OCT in routine clinical practice and determine its impact on patient outcomes.

##### Advances in OCT Technology

Improvements in imaging devices, including a higher resolution and faster acquisition rates, are expanding the capabilities of OCT in visualizing microstructural details. Additionally, the development of advanced image analysis algorithms and machine learning techniques has enabled automated identification and quantification of plaque features, reducing the subjectivity and time required for interpretation. Regardless, there is a need to develop a validated algorithm for plaque characterization that can help to facilitate the standardization of OCT image detection of plaque instability. This goal has been achieved by He et al., who designed a machine learning algorithm for the characterization of atherosclerotic plaque components by intravascular OCT using ex vivo carotid plaque tissue samples [47]. A total of 31 patients underwent carotid endarterectomy and the ex vivo carotid plaques were imaged with OCT. The algorithm was validated against histology slices, and it was capable of characterizing the fibrous, calcified, and lipid tissue of the carotid plaque with an excellent accuracy using the combined feature set [47].

### 2.3. Near-Infrared Spectroscopy (NIRS)

Near-infrared spectroscopy (NIRS) is a novel imaging technique that utilizes near-infrared light to analyze the absorption pattern of cholesterol molecules within the vessel wall, enabling the detection of lipid-rich plaques with high accuracy [48]. NIRS also provides valuable information about cerebral hemodynamic conditions and has the potential to serve as a brain monitor in various clinical scenarios, especially during carotid endarterectomy [49]. While commonly used as a non-invasive technique, a catheter-based NIRS proved to be accurate in detecting high lipid core plaque in atherosclerotic plaques. In 2002, Moreno et al. reported the successful application of NIRS in detecting lipid-rich necrotic cores (LRNC) in human aortic specimens. Histological analysis demonstrated NIRS’s high sensitivity and specificity, with values of 90% and 93% for identifying lipid pools, and 77% and 93% for identifying thin caps, respectively [48].

However, there are limitations that have hindered its independent use in clinical settings. Firstly, NIRS only provides information regarding the lipid composition of plaques and does not offer a comprehensive morphological assessment. Secondly, it cannot visualize or evaluate the size of the lumen, external vessel wall, or plaque burden. Lastly, NIRS lacks the depth resolution required to precisely locate the necrotic core within the plaque and differentiate TCFA from thick-cap fibroatheromas.

It is worth noting that NIRS provides limited anatomical information and is commonly used in conjunction with intravascular ultrasound (IVUS) to generate a “chemogram” or probability map. The chemogram represents the pullback position in millimeters on the *X*-axis and the circumferential position in degrees on the *Y*-axis, resembling the longitudinal splitting of the coronary vessel. In studies comparing NIRS to IVUS alone, NIRS has demonstrated superior performance in identifying lipid core plaques [50]. Some studies have explored the combination of NIRS with OCT probes to further enhance the system’s accuracy [51]. Its integration with other imaging modalities and further advancements in technology are expected to overcome its limitations and enhance its clinical utility in the future.

### 2.4. Hybrid Intravascular Imaging Modalities

Multimodal imaging approaches, combining different imaging modalities such as IVUS, OCT, and NIRS, have emerged to overcome the limitations of individual techniques and provide a comprehensive assessment of plaque morphology and composition as well as a prediction of disease progression. Studies have shown that a hybrid approach with IVUS and NIRS imaging is particularly advantageous in identifying the distribution of lipid core plaques and exploring the relationship between vascular geometry, shear stress, and plaque composition [52]. While IVUS alone can detect fibrous atherosclerotic plaques, which may be obscured by the presence of calcification, NIRS can detect lipids even in the presence of calcifications [53]. In the PACMANAMI randomized clinical trial, the combination of IVUS and NIRS was successfully utilized to evaluate the effect of statins on plaque burden and composition [54]. The ATHEROREMO-IVUS study and other recent prospective studies have suggested that IVUS-NIRS can serve as a diagnostic tool in clinical practice for detecting unstable plaques, especially fatty plaques, and identifying patients at high risk of subsequent major adverse cardiovascular events [55].

However, IVUS-NIRS also has limitations as the low resolution of IVUS affects the evaluation of cap thickness and luminal boundary definition in the presence of thrombosis or severe intraplaque bleeding. Alternatively, it has been suggested to integrate the two approaches of IVUS and OCT in order to benefit from the deep penetration of IVUS and the high resolution of OCT [25]. The application of IVUS-OCT has demonstrated improved imaging characteristics and provided supplementary information for detecting TCFA [56]. Furthermore, OCT-NIRS catheters have been developed to acquire OCT and NIRS data in a pull-back manner, combining the advantage of NIRS in identifying lipid core components with the advantage of OCT in determining fibrous cap thickness over lipid pools [57]. Additionally, other innovative multimodal imaging techniques such as OCT–near-infrared fluorescence (NIRF), IVUS–NIRF, IVUS–intravascular photoacoustic imaging (IVPA), and IVUS–fluorescence lifetime imaging microscopy (FLIM) are currently undergoing preclinical evaluation [58,59,60,61].

### 2.5. Carotid Angiography

Carotid angiography is considered to be the gold standard in evaluating carotid artery disease. In the North American Symptomatic Carotid Endarterectomy Trial (NASCET) and European Carotid Surgery Trial (ECST), angiography served as the reference standard for assessing luminal stenosis in carotid extracranial disease [62,63]. Based on these trials, stenosis emerged as a crucial factor in determining stroke risk.

In as early as 1978, Moore et al. noted that the presence of ulceration observed on angiography could identify patients at high risk of subsequent strokes [64]. In the initial 500 patients enrolled in NASCET, angiography was performed to detect ulceration and was subsequently compared to observations during endarterectomy. The sensitivity and specificity of angiography in detecting ulcerated plaques were 46% and 74%, respectively. The positive predictive value for identifying an ulcer was 72% [65]. A similar study design was employed in ECST, involving 1671 patients, with sensitivities and specificities for ulceration of 69% and 47%, respectively [66]. Studies that compared the radiological appearance with the histology of resected plaques showed a wide range of sensitivity and specificity, indicating substantial variability in the results [67,68,69]. Therefore, due to this variability, angiography provides little information regarding the actual risk of plaque instability.

## 3. Biomarkers of Non-Invasive Imaging

### 3.1. Carotid Ultrasound (US)

**Figure 3:**
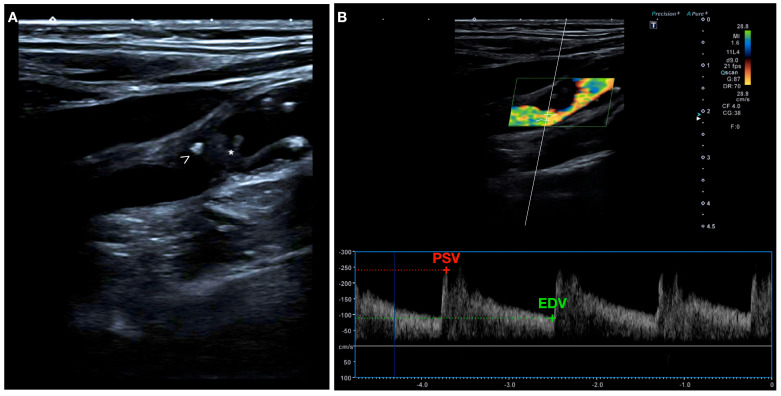
(**A**) B-mode ultrasound showing a longitudinal view of the internal carotid artery (ICA) with a heterogeneous echolucent plaque of irregular surface (*) and areas of focal calcification (arrow head). (**B**) High turbulence of the flow in the color Doppler study suggests the presence of severe stenosis. Spectral Doppler measurement of peak systolic velocity (PSV) = 245 cm/s, end diastolic velocity (EDV) = 90 cm/s, and ICA/carotid common artery (CCA) ratio = 3.7.

US has been widely used since the 1980s, particularly duplex ultrasonography, for quantifying the degree of carotid stenosis by measuring flow velocity and flow ratios, and it is the modality of choice for the initial evaluation of carotid artery disease [70,71]. In addition, it can also provide information on plaque instability based on the plaque surface and composition [72,73] (Figure 3).

#### 3.1.1. Fundamental Concepts and Methodological Approaches in US

**Figure 4:**
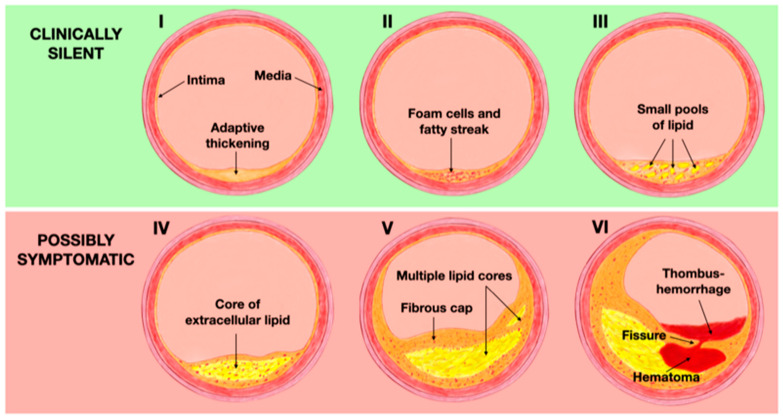
Histological classification and most common characteristics of AHA Type I to VI atherosclerotic plaques.

Originally, the appearance of a plaque was either classified as echogenic (calcified) or echolucent (non-calcified) [74]. Later, in an attempt to decrease observer variability, more detailed classifications were developed, such as the one proposed by Gray–Weale and Geroulakos [75]. Based on the echogenicity of the plaque, a five-category Gray–Weale scale was developed, from type I (uniformly echolucent) to type V (highly calcified) [75]. The Gray–Weale scale was used in the Tromsø Study, in which echolucent plaques were found to predict a higher risk of cerebrovascular events over a 3-year follow-up period [76]. However, these classifications exhibited weak inter-investigator reliability and little or no agreement with histologic results [77]. Regarding the histological features, the American Heart Association (AHA) introduced a well-validated classification system in 1995, that categorizes the distinct phases involved in the development and progression of atherosclerosis [78]. During the early stages of life, changes in the intima start with the deposition of macrophages, which later transform into foam cells as a result of excessive cholesterol phagocytosis. This process leads to the adaptive thickening of the intima, classified as Type I according to the AHA classification. These macrophages filled with lipids accumulate in multiple layers, resulting in the formation of fatty streak lesions (Type II). Type III plaques are characterized by the extracellular accumulation of lipids in small pools and can be considered as a pre-atheroma stage. These first three phases are clinically silent [79]. As the disease progresses, extracellular lipid merges into a dense lipid core within the intima, leading to the formation of atheroma lesions (Type IV). Macrophages, foam cells, and lymphocytes infiltrate the periphery of the lesion, while neovessels surround it, facing the lumen. The progression of Type IV lesions to Type V occurs when the lipid core becomes covered by a fibrotic layer, also known as a fibroatheroma (Type V). Type IV and V plaques are at risk of complications due to the appearance of fissures resulting from plaque disruption, intraplaque hemorrhage, or thrombosis, thus becoming Type VI or complicated lesions. This stage is considered to be the most advanced of the atherosclerotic process, often associated with significant stenosis or obstruction of the lumen and it is usually symptomatic due to the prothrombotic characteristics of the plaque that significantly increase the risk of ischemic events [78,79] (Figure 4).

In order to reconcile the discrepancies between ultrasound classification and histological findings, computer-assisted image analysis has been used to quantify plaque echogenicity. Nowadays, there are several possibilities for analyzing US images, such as the Gray Scale Median (GSM), Pixel Distribution Analysis (PDA), and Virtual Histology (VH).

GSM values are calculated by digitizing B-mode images and subsequently processing them with Adobe Photoshop (Adobe Systems Inc, San Jose, Calif). The Imaging in Carotid Angioplasty and Risk of Stroke study, a large-scale study that examined the relationship between GSM and the risk of stroke during carotid artery stenting, demonstrated that the rate of stroke and TIA was greater in patients with plaques that had GSM values of <25 than in patients with GSM > 25 [80].

Another approach is PDA, which maps individual tissue components within the carotid plaque image [81]. PDA digitizes ultrasound scan images and normalizes pixel intensities between two reference points (blood and arterial adventitia). This technique allows the application of a false color scale, creating a form of VH [81].

Despite the high expectations projected onto these imaging techniques, the results of the study conducted by Denzel et al. were not as encouraging. They compared B-mode images of 107 carotid endarterectomy specimens and their GSM values to a histologic classification consisting of only three groups (calcium-rich, lipid-rich, and combined plaques). Only 46% of the cases showed agreement between the GSM and the histopathological findings [82]. Correlation of PDA with histology showed similar results [12,81].

##### Three-Dimensional US (3D US)

Three-dimensional US provides better visualization of plaque geometry, surface irregularity, luminal plaque borders, intima-media layers, and ulceration compared to 2D ultrasound. It enables differentiation between ulceration and gaps between adjacent plaques, enhancing diagnostic accuracy [83], as well as quantification of plaque volume, which has been found to be a stronger predictor of coronary artery disease (CAD) compared to current 2D methods like intima-media thickness (IMT) measurement [84]. The presence and quantification of ulcers using 3D ultrasound in association with carotid stenosis have shown a correlation between the number of ulcers and the risk of stroke or death [85]. More recently, Muraki et al. demonstrated high sensitivity (85.7%) and specificity (81.3%) for the detection of plaque ulceration [86]. Kanber et al. developed an algorithm for detecting plaque surface irregularity using software to calculate the sum of the angular deviations of a plaque’s surface from a straight line, naming it the surface irregularity index (SII) [87]. The investigators found that the SII alone could predict the presence of cerebrovascular symptoms with a 66% accuracy and, in combination with stenosis, had an accuracy of 83% [87].

##### Contrast-Enhanced Ultrasound (CEUS)

Contrast-enhanced ultrasound (CEUS) is an innovative diagnostic tool that utilizes microbubble contrast agents to offer an objective evaluation of carotid plaque vulnerability. It allows for the visualization of neovessels within the plaque and plaque ulceration, assisting in distinguishing between occlusion and stenosis [88]. During the early phase of contrast administration, the neovessels fill with blood, leading to stronger echogenicity. In the late phase, JBA (defined as an area of pixels with a greyscale value < 25 adjacent to the lumen without a visible echogenic cap after image normalization) can be observed, indicating hypoechoic areas without a fibrous cap, and containing fragments of the lipid core in ruptured plaques [89]. A large JBA (>6 mm^2^) may indicate vulnerable plaques, while a discrete white area (DWA) is related to neovascularization [90].

Van den Oord et al. found that CEUS changed the risk category in asymptomatic patients previously classified by the traditional risk stratification model by calculating the Prospective Cardiovascular Munster Heart Study (PROCAM) risk [91]. Hamada et al. validated plaque ulceration assessed by CEUS with histology analysis, confirming its high sensitivity for identifying plaque ulceration and fibrous cap disruption [92].

#### 3.1.2. Features of a Vulnerable Carotid Plaque: Insights from US Imaging

##### Plaque Composition

The TCFA can be visualized on ultrasound as an echogenic structure that reflects more echoes than the surrounding plaque and blood [71]. The thickness of the TCFA can be measured by using stratified GSM measurements, which have a sensitivity of 73% and specificity of 67% [93]. The lipid-rich necrotic core or IPH of a vulnerable plaque may be identified by assessing echo intensity [84]. On US, lipid appears as an echolucent area that looks similar to IPH. The presence of lipid necrosis can cause the plaque to have a heterogeneous appearance [94]. The Asymptomatic Carotid Stenosis and Risk of Stroke (ACSRS) study used the JBA to refer to either IPH or a large fatty core and found that this feature was associated with an increased risk of stroke [95]. The size of the lipid core is believed to be a critical factor in plaque stability, with larger pools of lipid being associated with less stable plaques [96]. However, data on the ability to detect the lipid core using ultrasound or computer-aided greyscale analysis are conflicting [81,97]. Calcification appears as a bright or hyperechogenic area on ultrasound and it can be assessed by the mean pixel value [98], GSM [99], and PDA [81]. One limitation of assessing heavily calcified plaques with ultrasound is that the shadowing effect can limit the assessment of other plaque characteristics and the severity of stenosis. The presence of a thrombus or intraplaque hemorrhage can cause an echolucent lesion that resembles a fatty core [71]. Conventional 2D ultrasound has been found to have a high level of accuracy in detecting an intraluminal thrombus, with sensitivity and specificity ranging from 80% to 90% and 80% to 91%, respectively [100]. In contrast, the findings from the GSM measurement and histology of IPH have been found to be less-well correlated [101].

##### Plaque Morphology

On ultrasound, ulceration is defined as a focal depression of at least 2 mm deep and 2 mm long, with a distinct wall at its base, and a region of reversed flow at the site of the recess [83,102]. However, when CEUS is performed, ulceration is defined as a plaque–lumen border disruption filled with microbubbles and measuring at least 1 × 1 mm [103]. In many instances, the varying sensitivity (33–75%) and specificity (33–92%) of ultrasound in detecting plaque ulceration may be attributed to a lack of experience in identifying this feature [104,105].

##### Plaque Activity

Plaques with higher enhancement have been correlated with a greater neovascularization on histology analysis [106]. Camps-Renom et al. demonstrated that plaque neovascularization detected by CEUS in patients with anterior circulation ischemic stroke and carotid atherosclerosis was an independent predictor of stroke recurrence [107].

#### 3.1.3. Clinical Practice Perspectives and Emerging Trends in US

##### Diagnostic and Therapeutic Implications

US allows for the measurement of flow velocity, detection of stenosis severity, and visualization of plaque echogenicity. It can also provide information on plaque vulnerability based on the plaque surface and composition [72,73]. Plaque activity can also be estimated by CEUS. Van Engelen et al. demonstrated that changes in carotid plaque texture and total plaque volume predicted cardiovascular events in subjects with increased Framingham risk scores, suggesting the potential for integrating texture analysis to enhance risk stratification [108].

In addition, the development of computer-assisted image analysis has reduced inter-observer variability in ultrasound scan analysis. The Imaging in Carotid Angioplasty and Risk of Stroke (ICAROS) trial demonstrated that increased echolucency of the carotid plaque, measured by GSM, is a risk factor for stroke during and immediately after carotid artery stenting [109]. However, since the ICAROS trial focused on patients receiving a carotid stent, a histological substrate was not available for comparison with GSM values [109].

##### Advances in US Technology

Recent advancements in integrated backscattering (IB) analysis have created a map-like image that describes the mixed composition of the plaque, providing more detailed information than averaged IB values. Custom software, such as iPlaque, allows the visualization of different tissue components of a plaque in a color-coded display, based on previously established IB threshold values [110].

A limitation of GSM analysis is that by analyzing the median gray value of the plaque as a whole, it may overlook significant heterogeneity within the plaque and misrepresent instability if a plaque contains both soft and hard components. Texture analysis is an alternative approach that takes into consideration the heterogeneity and spatial variations in pixel intensity within the plaque. Acharya et al. used semi-automatic texture features and features based on a trace transform matrix to classify plaques as symptomatic or asymptomatic, achieving high accuracy rates [111].

While the widespread applicability of these methods is still limited, recent studies have suggested that the grayscale algorithms used for two-dimensional (2D) images can be applied to three-dimensional (3D) ultrasound for whole-plaque analysis [112]. The combination of GSM and texture analysis in 3D ultrasound may enable the identification of unstable regions within a plaque, but further validation of this method is needed [83,112].

### 3.2. Transcranial Doppler Ultrasonography

Transcranial Doppler ultrasonography (TCD) is a non-invasive and portable imaging technique that is used to visualize the intracranial blood vessels. To perform imaging, a sector-array transducer with a low-frequency (2 MHz) is typically used to allow the signal to penetrate through the skull.

The primary role of TCD in carotid imaging is to evaluate cerebral microembolic signals (MESs), and the middle cerebral artery is the preferred artery to monitor [113]. In patients with symptomatic carotid stenosis, MESs were found in 43% of patients, compared to just 10% in asymptomatic patients, as reported by Ritter et al. [114]. Conversely, the absence of MESs indicates a very low risk of future symptoms in patients with asymptomatic carotid plaques. This is further supported by the fact that MESs rapidly diminish following CEA [115].

Additionally, TCD is useful in assessing the risk of stroke in these patients, especially when combined with other imaging modalities. The combination of plaque echolucency on B-mode ultrasonography and MESs on TCD has been associated with a 10-times-greater risk of stroke in patients with asymptomatic carotid stenosis [116]. The combination of plaque neovascularization on contrast-enhanced ultrasound (CEUS) and MESs on TCD is also emerging as a strong risk factor for acute ischemic stroke [117].

### 3.3. Elastography

Elastography is a valuable technique for evaluating the stiffness of a plaque, which reflects its histological composition [118]. By measuring plaque displacement and deformation, elastography assesses the mechanical properties of the tissue.

Two methods of elastography are commonly used: strain elastography (SE) and shear-wave elastography (SWE). SE measures the displacement of the plaque caused by external forces such as blood pressure oscillations or manual compression of the probe and provides semi-quantitative parameters like strain, strain velocity, or strain rate using deformation estimating algorithms. On the other hand, SWE involves the emission of shear waves into the tissue through an acoustic radiation force impulse. These waves propagate perpendicularly to the impulse, and the technique measures their velocity, expressed as Young’s modulus (YM). YM defines tissue resistance to elastic deformation and quantifies the stress required to achieve a unit of deformation, thus providing a measure of tissue elasticity [118].

Plaques with a higher lipid content exhibit significant elastic deformation, a lower YM, and lower shear-wave velocities (SWV). In contrast, more rigid tissues like calcified plaques demonstrate less elastic deformation and higher SWV [119]. A lower mean YM and SWV were found in symptomatic plaques compared to the asymptomatic group [120]. SWV were also shown to be lower in hypoechoic plaques, suggesting that SWE indices could be used to differentiate vulnerable from less vulnerable plaques. SWE imaging has demonstrated its value in identifying carotid plaques prone to rupture by correlating YM values with the Gray–Weale echogenicity grading and GSM values [121]. YM was found to be a superior vulnerability marker compared to GSM, and combining YM values with the degree of stenosis improved diagnostic performance [120]. Studies have shown increased SWE displacements in regions identified as lipid on MRI, and larger local deformations and increased complexity in deformation patterns are more likely to occur in vulnerable plaques [122,123].

The sensitivity and specificity of elastography vary depending on the reference method used. When compared to MRI, sensitivity was reported as 71.4%, and specificity was 87.1%. In comparison to histology, sensitivity decreased to 50%, while specificity reached 100% [124].

While elastography indices cannot replace the grading of stenosis for determining eligibility for surgery, they can provide additional information to improve the detection of unstable plaques and patient risk stratification. However, further large-scale studies with longitudinal follow-up are warranted to enhance our understanding of this technique.

### 3.4. Computational Fluid Dynamics (CFD)

Computational fluid dynamics (CFD) technologies are used to analyze and quantify fluid flow behavior in various systems, including the cardiovascular system. In the context of atherosclerotic plaques, CFD allows for the calculation and visualization of hemodynamic forces acting on the plaque, such as wall shear stress (WSS) and axial plaque stress (APS). These technologies have undergone significant progress, now enabling the utilization of more accurate and patient-specific geometric models derived from sources like coronary computed tomography angiography (CTA). By discretizing the coronary models into volumetric meshes, CFD analysis can simulate blood flow and pressure patterns, providing valuable insights into the behavior of atherosclerotic plaques and their susceptibility to rupture or complications [125].

WSS is a critical factor in the development and rupture of atherosclerotic plaques. High WSS has been observed to co-occur with plaque rupture in various artery imaging studies. However, solely considering WSS magnitude may not fully predict the rupture process. To address this, researchers have introduced APS as an alternative measure to estimate plaque rupture risk. APS has shown statistical associations with necrotic-core plaques and functional ischemia in the coronary arteries [125,126].

Li et al. established a clear association between plaque geometry and stress, as measured by WSS and APS. Plaque severity and eccentricity were identified as independent factors linked to acute vascular events in coronary plaques, while the lengths of proximal and distal segments indicated potential sites of rupture. APS was found to be directly linked to plaque rupture, especially in the distal segments [126]. Another study by Choi et al. revealed distinct APS distributions between upstream-dominant and downstream-dominant lesions, indicating a lower risk of downstream rupture in cases of severe stenosis due to decreased downstream pressure. Moreover, a significant negative correlation between APS and lesion length can provide an explanation for the higher incidence of plaque rupture in short and focal lesions compared to diffuse lesions [125].

The enhanced capabilities of these hemodynamic and geometric indices have opened up new possibilities for studying plaque-related pathologies and guiding personalized treatment strategies.

### 3.5. Carotid Computed Tomography (CT)

**Figure 5:**
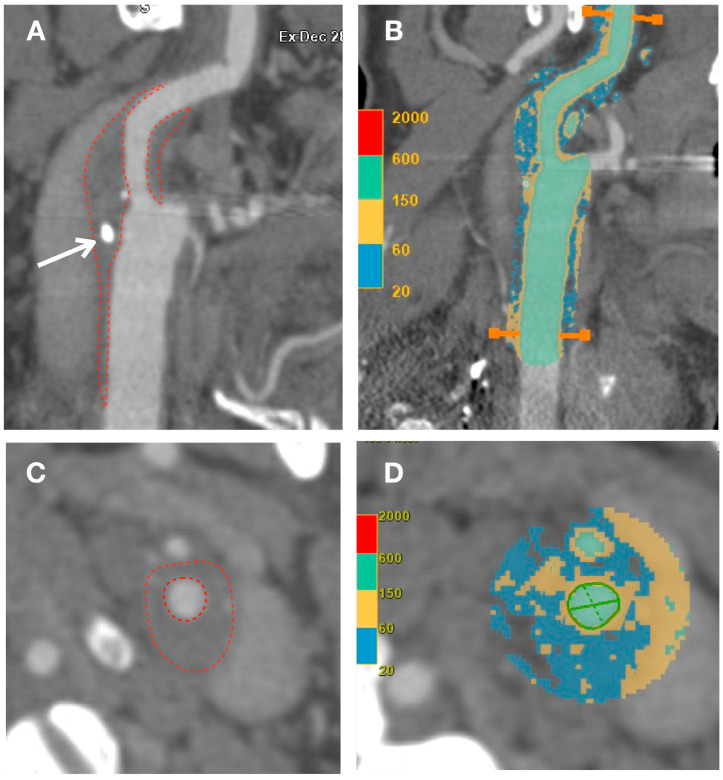
CT imaging can be used for the analysis of carotid plaque by assessing the density of its components, which is measured in Hounsfield units (HU). Image (**A**) shows a sagittal view of CCA and ICA with a low-density plaque (red lines) and spotty calcification (white arrow). Image (**C**) displays an axial view of the ICA. Images (**B**) (sagittal view) and (**D**) (axial view) represent the automated color coding of carotid plaque components. The predominant color is blue, which correlates with a lipidic core (<60 HU).

Computed tomography (CT) is a non-invasive imaging modality that has proven to be an excellent tool for carotid stenosis evaluation and it also provides a viable alternative for assessing vessel wall size, high-risk plaque burden, morphological characteristics, and vulnerability, with a relatively high accuracy [127] (Figure 5).

#### 3.5.1. Fundamental Concepts and Methodological Approaches in CT

There are two primary CT techniques used for plaque characterization: multidetector-row CT angiography (MDCTA) and dual-source CT (DSCT). MDCTA allows for reconstructions in multiple planes (axial, sagittal, and coronal) and provides high spatial and contrast resolution, similar to MRI [128]. MDCTA has demonstrated excellent sensitivity and specificity in detecting plaque ulcers and plaque neovascularization, both surpassing 90% [129].

DSCT utilizes two different X-ray sources operating at different energies to achieve distinct Hounsfield units (HUs) within the same tissue. This allows for improved tissue differentiation and advanced postprocessing techniques. DSCT also can be combined with bone-removal algorithms, enabling better visualization of the vasculature and providing a high spatial resolution for multiplanar reformats [130]. Compared to standard MDCT, DSCT has advantages such as the ability to differentiate calcified plaque from iodinated contrast, which facilitates the accurate assessment of calcified plaque volume and easy bone subtraction [130].

#### 3.5.2. Features of Vulnerable Carotid Plaque: Insights from CT Imaging

##### Plaque Composition

As a general guideline, lower plaque density is indicative of increased instability [128]. The thickness of the TFCA can be measured with a good correlation to histology, and MDCTA has shown an association between a fissured fibrous cap and cerebrovascular symptoms [131]. IPH is characterized by low HU and Saba et al. [132] proposed a <25 HU threshold, but this is still vastly debated in the literature. A lipid core can be detected as an area of lower density. Multidetector computed tomography (MDCT) has shown good correlations with histology in identifying large lipid cores, although this is limited to mildly calcified plaques. Distinguishing IPH from lipid-rich non-calcified components on CT is a nontrivial challenge because both IPH and LRNC have low overlapping CT numbers of <60 HU [131]. MDCT can also accurately detect the presence and amount of calcification due to their high density. In fact, CT is considered to be the most effective imaging technique for identifying calcification in carotid plaques [133]. On CT, a soft plaque is generally defined as a low-attenuation plaque with approximately <60 HU, whereas fibrous tissue is considered to be between 60–130 HU and >130 HU is considered to be a calcified plaque [134]. However, there is significant overlap in HU values among LRNC, connective tissue, and IPH, and the presence of calcification artifact limits its usefulness in plaque analysis [131,135]. In this regard, it should be noted that, regarding the extraction of calcified plaques, de-blooming algorithms have been effective in minimizing blooming artifacts for calcified plaque extraction, but further investigation is needed to address other factors affecting accuracy and reliability, such as individual differences and co-existing plaques and stents [136].

##### Plaque Morphology

A plaque ulcer is characterized by the presence of an intimal defect that causes the contrast material to extend beyond the lumen and into the surrounding plaque on CT imaging. MDCTA can detect ulceration with moderate to good sensitivity (60–94%) and specificity (70–99%) when compared to histological analysis [104,137].

##### Plaque Activity

CT can also identify and quantify neovascularization as well as plaque volume and vascular remodeling [90,132]. In a study of 97 patients, Saba et al. found that symptomatic plaques exhibited significantly higher degrees of plaque enhancement following contrast administration compared with asymptomatic plaques. A threshold of 15 HU had a specificity of 83% and a sensitivity of 76% [128]. Delayed-phase images have also demonstrated a strong correlation with symptomatology, with stable plaques exhibiting progressive enhancement on delayed images, while symptomatic plaques tend to show more washout. This is likely due to the presence of neovascularization within unstable plaques, leading to increased contrast washout on delayed images [138].

#### 3.5.3. Clinical Practice Perspectives and Emerging Trends in CT

##### Diagnostic and Therapeutic Implications

MDCTA is already integrated as standard care for the evaluation of carotid plaques. Observation of the characteristics of plaque instability in CT studies would greatly reduce the need for further imaging assessment. However, stratification tools for vulnerable carotid plaque diagnosis in CT studies have yet to be developed, as the literature lacks comparative studies with systematic reporting of outcomes between the two imaging modalities. In particular, MDCTA has been used to monitor the effects of statins, showing modifications in plaque composition over time, with a progressive reduction in fatty subcomponents [34,139]. Additionally, it has been demonstrated that the identification of high-risk markers of carotid atherosclerosis in MDCTA can predict 10-year atherosclerotic cardiovascular disease risk scores [140].

##### Advances in CT Technology

A study by Ball et al. utilized a novel technology called tomographic ultrasound (tUS) [141]. This technology involves a three-dimensional (3D) ultrasound system with a spatial tracker that computes multiplanar reconstructions to produce 3D ultrasound volumes. The study found that tUS is an accurate method that offers all the advantages of ultrasound [89].

Other recent studies suggest that high shear stress contributes to the progression of unstable plaques [142], but little is known about the exact pathophysiological mechanism of shear stress in plaque progression. MDCTA 3D lumen geometry assessment may contribute to a better understanding of various hemodynamic factors, including shear stress, in the future [143].

### 3.6. Carotid Magnetic Resonance Imaging (MRI)

**Figure 6:**
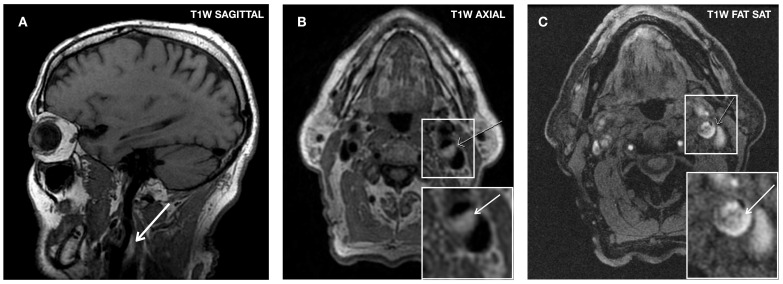
MRI images of an IPH. T1-weighted (T1W) sagittal (**A**) and axial views (**B**) show hyperintense signal within the carotid plaque (white arrows). On the T1W with fat saturation image (**C**) the plaque also presents areas of high intensity, indicating the presence of IPH.

Histological correlation studies have demonstrated the high sensitivity and specificity of in vivo high-resolution MRI in identifying vulnerable plaque characteristics [144]. The best potential of MRI lies in estimating the thickness of the fibrous cap and detecting the presence of IPH, being particularly adept at distinguishing between LRNC and IPH [32]. MRI has proven clinically valuable in detecting IPH with a sensitivity ranging from 82% to 97% and a specificity of 74% to 100% [145,146] (Figure 6).

#### 3.6.1. Fundamental Concepts and Methodological Approaches in MRI

There are multiple pulse sequences available for characterizing plaques using MRI. One frequently utilized technique is rapid spin echo (RSE), which enables imaging with T1-weighting, T2-weighting, and proton density weighting (PDW) [147].

Magnetization-prepared rapid-acquisition gradient echo (MPRAGE) combines the use of magnetization preparation with rapid image acquisition using gradient echoes and it reliably detects IPH and LRNC on T1-weighted images [148].

The black-blood technique is commonly employed for plaque imaging. It employs an RSE sequence with double inversion recovery preparatory pulses, enhancing the contrast between the dark lumen and the vessel wall. However, this technique often necessitates longer examination times [149].

To accurately characterize plaque morphology, fat suppression is critical. This technique is utilized in all sequences to suppress the signal from the subcutaneous fat, improving the contrast between different plaque components as well as between the carotid wall and surrounding tissues. Fat-suppressed T1-weighted images are particularly valuable in differentiating the high T1 signal of intraplaque lipid from that of IPH [150].

Contrast-enhanced images play a pivotal role in distinguishing various plaque components. Gadolinium (Gd)-based contrast imaging can be employed to evaluate plaque neovascularity and differentiate a necrotic core from fibrous tissue on T1-weighted images [151,152].

#### 3.6.2. Features of Vulnerable Carotid Plaque: Insights from MRI Imaging

##### Plaque Composition

On MRI, the fibrous cap can be visualized as a thin band adjacent to the lumen. It appears hypointense on time-of-flight (TOF)-weighted images and isointense on T1-weighted (T1W), T2-weighted (T2W), and proton-density-weighted (PDW) images. A ruptured FC will demonstrate a disrupted, dark band on CE-T1W with an irregular luminal surface on all images, although distinguishing between the two is still challenging [145]. Using 3D TOF MRA, Hatsukami et al. demonstrated a high level of agreement (89%) between MRI and histological findings [153]. The necrotic core and IPH do not exhibit enhancement as they lack vascularity, whereas the fibrous cap component of the plaque demonstrates enhancement. Increased enhancement with Gd is also associated with neovascularity and plaque inflammation [151,152]. LRNC is hypointense on T2W images and will not enhance on CE-T1W images. The detection rate of an LRNC is slightly better when IPH is not present [154]. Differentiating IPH from LRNC can be challenging as the thrombus is often located within the necrotic core. IPH typically appears hyperintense on all T1W imaging sequences while LRNC appears hyperintense only on T1-weighted images and isointense on TOF MR images [154] IPH was found to be detectable with both T1W and TOF MRA images at 1.5 T with a sensitivity of 82% and specificity of 77% by Saam et al. [155], whereas 3T MPRAGE depicted IPH with a similar sensitivity (80%) but a much higher specificity (97%) [156]. MPRAGE, as compared with FSE and TOF, demonstrated higher diagnostic capability for the detection and quantification of IPH [156]. MRI can detect calcification in the vessel wall with a sensitivity ranging from 76% to 84% and a specificity ranging from 86% to 94%. Calcification appears hypointense on all contrast images [145,149,154]. However, measuring the area of calcification as a percentage of the vessel wall using histology as the reference may lead to underestimation [149].

##### Plaque Morphology

MRI identifies ulceration using a variety of imaging sequences such as 3D TOF, T1, proton density, T2, and contrast-enhanced T1 [157]. In MRI the fibrous cap appears as a dark band between the bright lumen and the gray plaque. The absence of this thin dark band indicates plaque ulceration on all contrast weightings. The sensitivity and specificity of MRI in identifying ulceration can be further improved by incorporating longitudinal black-blood MR angiography, which results in a sensitivity and specificity of 80% and 70%, respectively [158].

##### Plaque Activity

Plaque enhancement on post-contrast T1-W MR images is associated with plaque vulnerability, neovascularization, and macrophage infiltration [79]. Dynamic contrast-enhanced (DCE) MRI has been employed in previous studies to quantify plaque enhancement, neovascularity, and inflammation [151,159], demonstrating a significant association between plaque enhancement and ipsilateral ischemic events, independent of the degree of stenosis [144]. Millon et al. found that neovascularity was observed in up to 97% of areas with gadolinium (Gd) enhancement on post-contrast images, while macrophage infiltration was seen in 87% of regions of Gd enhancement [144].

#### 3.6.3. Clinical Practice Perspectives and Emerging Trends in MRI

##### Diagnostic and Therapeutic Implications

MRI is currently the most promising imaging modality for identifying vulnerable plaque components, thanks to its high soft-tissue contrast and high in-plane resolution. Studies have shown that MRI can detect most of the described plaque characteristics with moderate to good agreement [145,146,154,155,158].

Studies involving both asymptomatic and symptomatic patients with moderate carotid stenosis (<70%) have shown that MRI findings of IPH are associated with a higher hazard ratio for future ipsilateral ischemic events [79,144,155,160]. There exists a subpopulation of clinically stable asymptomatic patients with cardiovascular disease whose carotid plaque contains IPH despite maximum-tolerated intensive statin therapy. MRI can reclassify these patients without clinical very-high-risk features into an imaging-defined very-high-risk group who may benefit from very intensive lipid-lowering therapy [161]. However, there are still potential obstacles to its widespread adoption as a routine risk stratification tool. The associated high cost and limited availability are the most significant barriers.

##### Advances in MRI Technology

There is significant interest and ongoing research regarding potential biomarkers that are specifically designed to target molecules present in unstable atherosclerotic lesions. Superparamagnetic iron oxide (SPIO) particles have become the favored MR contrast agent that targets specific molecules or cells such as elastin, fibrin, or vascular cell adhesion molecule 1 [162]. These particles induce magnetic susceptibility on T2-weighted images and are useful for identifying plaque macrophages as surrogate markers of plaque inflammation in the assessment of instability of carotid plaques [163].

One difficulty with multi-contrast carotid plaque MRI is needed to co-register multiple sequences. A newly developed 3D sequence to obtain three different contrast weightings (T1, T2, and gray blood) during a single 5-min acquisition can streamline carotid plaque imaging and analysis. The multi-contrast atherosclerosis characterization (MATCH) sequence was used in 53 consecutive patients, and it was comparable, if not superior, to conventional multi-contrast carotid plaque MRI in identifying and quantifying major carotid plaque components [164]. In another effort to streamline carotid plaque compositional analysis, the simultaneous non-contrast angiography and intraplaque hemorrhage (SNAP) technique allows for imaging of the blood vessels without the need for contrast administration, while simultaneously identifying the presence of IPH in the carotid plaque. By combining these sequences, the carotid arteries can be visualized without the use of contrast agents, while also identifying areas of hemorrhage within the plaque [165].

Another area of future research could focus on the correlation between carotid plaque characteristics and cerebral damage in patients with silent cerebral ischemic events who are at high risk of a future stroke. The 7-Tesla MRI has proven to be superior in visualizing cerebral microbleeds and microinfarcts in symptomatic patients with high-grade stenosis than 1.5-Tesla and 3-Tesla [166].

However, the current manual processing of atherosclerotic plaque features derived from MRI wall imaging data is subject to inter- and intra-observer variability and is a time-consuming process. Therefore, automated segmentation techniques have been developed to overcome these limitations [143].

### 3.7. Positron Emission Tomography (PET)

Positron emission tomography (PET) is a medical imaging technique that uses radioactive tracers to assess the biological processes related to atherosclerosis, such as intraplaque inflammation, microcalcification, and intraplaque angiogenesis. Moreover, 18F-fluorodeoxyglucose (18F-FDG) is currently the most validated tracer for imaging plaque inflammation, particularly in vulnerable carotid plaques, where high FDG uptake has been shown to correlate with macrophage accumulation [167]. In addition to glucose uptake, nuclear imaging can target numerous other metabolic and signaling pathways associated with vulnerable plaques, such as low-density lipoproteins, matrix metalloproteinase inhibitors [168], and chemotactic proteins [32].

#### 3.7.1. Fundamental Concepts and Methodological Approaches in PET

The primary molecular imaging technique used for assessing carotid plaques is 18F-fluorodeoxyglucose (FDG) PET/CT. FDG is partially metabolized through glycolysis within the atherosclerotic plaque, and serves as an indicator of plaque inflammation and hypoxia [169]. Intravenous injection of FDG is followed by image acquisition after 60–180 min using a dedicated PET/CT scanner [170].

Tawakol et al. were the first to identify a histological association between plaque inflammation and the degree of 18F-FDG uptake [167]. The abundance of inflammatory cells is observed in highly inflamed vulnerable plaques, which take up FDG, a glucose analog. Higher metabolic activity leads to a more significant accumulation of FDG [89,171].

Special consideration is needed when utilizing FDG-PET/CT for plaque characterization. The concentration of FDG in the blood pool can affect contrast resolution, particularly when evaluating small areas like carotid plaques. The optimal timing for imaging following FDG administration is a subject of debate. The carotid maximal standardized uptake value (SUVmax) at 180 min is more strongly associated with a 10-year risk of fatal cardiovascular disease compared to imaging at 90 min, as well as the quantification of atherosclerotic plaque inflammation [172].

#### 3.7.2. Features of Vulnerable Carotid Plaque: Insights from PET Imaging

##### Plaque Activity

Researchers have explored the combination of FDG-PET to detect active inflammation and MRI to identify morphological features indicative of higher risk, aiming to optimize risk stratification. Truijman et al. found only a weak correlation between plaque inflammation on PET and neovascularization detected by dynamic contrast-enhanced (DCE) MRI, suggesting the complementary nature of these two techniques [173]. Calcagno et al. also identified a weak inverse relationship between neovascularization on DCE MRI and plaque inflammation on PET [174]. Similarly, FDG uptake did not strongly correlate with IPH observed on MRI [171]. In their examination of carotid artery specimens, Joshi et al. observed that 18F-NaF uptake occurred precisely at the site of all carotid plaque ruptures and was strongly associated with active calcification, macrophage infiltration, apoptosis, and necrosis [175].

In a study of patients undergoing carotid endarterectomy (CEA) after FDG-PET/CT, SUVmax was associated with an increased CD68 concentration, a marker of macrophage activity [176]. Tawakol et al. also found higher CD68 staining in plaques with high SUV compared to those with low SUV [167].

#### 3.7.3. Clinical Practice and Emerging Trends in PET

##### Diagnostic and Therapeutic Implications

PET is a validated imaging technique for plaque inflammation-related metabolism and plaque instability [177]. Additionally, FDG-PET can reveal common cardiovascular risk factors, showing significant correlations with factors such as obesity, male gender, age (>65 years), smoking, hypertension, diabetes, and hypercholesterolemia. These risk factors are associated with local arterial inflammation, highlighting the potential predictive value of FDG-PET imaging in disease progression [178].

Several studies have also reported a link between FDG uptake and the risk of future events in both asymptomatic and symptomatic individuals [167]. Skagen et al. showed that there was a greater uptake of 18F-FDG on PET/CT in patients with symptomatic carotid artery plaques compared with those who were asymptomatic [179]. In addition, Fujimoto et al. demonstrated that the uptake of 18F-NaF was related to the severity of ischemic vascular brain disease on MRI, which suggests that it may be useful in the risk assessment of cerebrovascular disease [180]. In this way, the Biomarkers Imaging Vulnerable Atherosclerosis in Symptomatic Carotid disease (BIOVASC) trial, involving patients with carotid stenosis and a recent stroke/transient ischemic attack, found that in patients with recent symptomatic carotid stenosis, plaque 18F-FDG uptake was associated with early recurrent stroke [181]. The study showed for the first time that plaque FDG uptake independently predicts early stroke after PET. This finding suggests that higher plaque FDG uptake is a marker of a vulnerable carotid plaque leading to stroke recurrence. These results led to the development of a novel score called the Symptomatic Carotid Atheroma Inflammation Lumen Stenosis Score (SCAIL) [182]. This score, developed by Kelly PJ et al., assigns points based on the severity of stenosis and 18F-fluorodeoxyglucose (18F-FDG) uptake and predicts the risk of recurrent ischemic stroke using 18F-FDG standardized uptake values on PET-CT as a parameter for plaque inflammation [182].

##### Advances in PET Technology

The development of novel PET tracers is an active area of extensive research. Some of these novel tracers, including 18F-FMISO, 68Ga-NOTA-RGD, and 18F-NAF, are being studied in humans as alternative markers of inflammation and potential markers for microcalcification, respectively, with some success [175,183,184]. Furthermore, 18F-NAF specifically targets active microcalcification in atherosclerotic plaques, which could be valuable in identifying vulnerable carotid plaques. Recent clinical trials have demonstrated that 18F-FMISO and 68Ga-NOTA-RGD are associated with angiogenesis and they have been proposed to target molecules that are highly expressed within vulnerable plaques [185,186,187]. Imaging of matrix metalloproteinases, proteases associated with plaque rupture, has also exhibited promise in early preclinical studies [188]. However, further validation through large-scale clinical studies is necessary for these radioactive tracers.

In terms of the translation of promising PET tracers into the clinical setting, the co-registration of PET images with CT or MRI PET has limited spatial resolution (3–5 mm), which restricts the direct quantification of vulnerable plaques in smaller vessels. To address this issue, hybrid scanner constructs, such as PET/CT and PET/MRI, are essential, with MRI potentially providing additional benefit over CT as it provides better visualization of the vessel wall [71].

## 4. Evaluating Imaging Modalities: A Comparative Approach

Although computed tomography (CT), magnetic resonance imaging (MRI), and sonography have demonstrated their effectiveness in detecting key markers of carotid plaque vulnerability, they have certain limitations in capturing cellular markers at a microscopic scale. In this context, optical coherence tomography (OCT) emerges as a promising and dynamic imaging-based modality for the real-time visualization of microvascular structures. OCT offers an impressive spatial resolution of around 10 μm in tissue [189], which is approximately ten times higher than IVUS [53]. This makes OCT well-suited for investigating plaque microstructure, fibrous cap thickness, and lipid-rich regions, which are crucial indicators of plaque instability [41]. Moreover, OCT provides imaging quality comparable to histological biopsy. On the other hand, IVUS has a lower spatial resolution compared to OCT but provides deeper penetration into the tissues. Therefore, IVUS is effective in visualizing plaque burden, identifying calcium deposits, and assessing vessel dimensions (Table 1).

One common misconception regarding OCT is that it can replace intravascular ultrasound (IVUS) [41]. In fact, each technology excels at different tasks, and it is important to highlight that both IVUS and OCT have their specific strengths and limitations, and a combined approach may be necessary for a comprehensive evaluation of the vulnerability of the carotid plaque.

CTA and MRA have been the cornerstones, along with US for the imaging of carotid artery disease. Arteriography has been largely sidetracked in recent decades due to its invasive nature and associated risks of complications. CTA has demonstrated its effectiveness in evaluating stenosis, while MRA offers detailed information about the morphology of atherosclerotic carotid lesions. MRA studies have demonstrated superior ability in distinguishing vulnerable carotid plaque characteristics such as TFCA, LRNC, IPH, plaque ulceration, and neovascularization [190]. Although CTA shows promising potential in detecting certain vulnerable lesions, limited data are available regarding these characteristics in CTA imaging sequences (Table 2).

## 5. Conclusions

Several imaging biomarkers have been identified for carotid plaque instability including intraplaque hemorrhage, neovascularization, plaque rupture, lipid-rich necrotic core, and fibrous cap thickness.

The clinical relevance of these imaging biomarkers lies in their ability to predict stroke risk and guide treatment decisions. Identifying vulnerable plaques with imaging biomarkers can aid in the selection of patients who may benefit from carotid endarterectomy or stenting. Additionally, monitoring changes in imaging biomarkers over time can help to assess the effectiveness of medical therapy or lifestyle modifications aimed at reducing stroke risk.

These biomarkers can be assessed using various non-invasive imaging modalities such as US, CT, MRI, and PET. US is commonly used as the primary imaging modality for initial evaluation, providing valuable information on flow velocity, stenosis severity, and plaque surface and composition. The implementation of 3D US enables the quantification of plaque volume and differentiation between ulceration and gaps within plaques; however, for the more precise assessment of ulceration, CEUS is preferred, which also allows visualization of neovessels. CT imaging is highly effective in accurately detecting calcification, and assessing vessel wall size, plaque burden, and morphological characteristics. MRI has demonstrated clinical significance in detecting IPH with high sensitivity and specificity. Lastly, PET currently stands as the most validated tracer for imaging plaque inflammation. In recent years, technological advancements have greatly contributed to the development of intravascular imaging techniques. The implementation of IVUS, OCT, and NIRS in the assessment of carotid plaque instability represents a significant improvement, with OCT showing the potential to achieve results comparable to those obtained through histological biopsy. However, considering their invasive approach, the use of these techniques in assessing risk in asymptomatic patients is highly debatable.

Incorporating a synergistic approach involving multiple imaging modalities is imperative in order to acquire a comprehensive understanding of the features and behavior of unstable plaques, thereby leading to a reduction in the risk of cerebrovascular events.

## Figures and Tables

**Table 1:** Comparison of intravascular imaging modalities for visualization of vulnerable plaque.

	IVUS	OCT	NIRS-IVUS	Angiography	US	CEUS	CT	MRI	PET
TCFA	-	+++	-	+	+	+	+	++	-
LRNC	+	+++	+++	-	-	+	+	++	-
IPH	+	+++	+++	-	+	++	++	+++	-
Calcification	+++	+++	+++	++	++	++	+++	++	-
Ulceration	+++	+++	+++	++	++	+++	+++	+++	-
Neovascularization	-	+	-	-	-	++	+	+	-
Inflamation	+	+	+	-	-	++	++	++	+++
Positive remodeling	+++	+	+++	-	-	++	++	++	+

Indicator: - means not detectable; + barely detectable; ++ visible; +++ well delineated.

**Table 2:** Advantages, disadvantages, and application scenarios of the various imaging techniques.

Imaging Technique	Advantages	Disadvantages	Application Scenarios
IVUS	High penetration	Invasive; spatial resolution; availability; cost	Calcification; ulceration; positive remodeling
OCT	High spatial resolution	Invasive; availability; cost	Hemorrage; Lipid component; calcification; ulceration
NIRS-IVUS	High penetration; high spatial resolution	Invasive; spatial resolution; availability; cost	Positive remodeling; hemorrage; Lipid component; calcification; ulceration
Angiography	-	Invasive; radiation; contrast; resolution; availability	Luminal stenosis
US	Noninvasive; radiation-free; wide availability; low cost	Operator dependency; variability; resolution	Ulceration
CEUS	Noninvasive; radiation-free; good availability; limited cost	Operator dependency; variability; resolution	Ulceration; neovascularization
CT	Noninvasive; high resolution; reproducibility	Radiation; contrast agents; calcification	Calcification; ulceration;
MRI	Noninvasive; radiation-free; high resolution; reproducibility	Gadolinium; costs; time; availability	Hemorrage; ulceration, necrosis,
PET	Noninvasive; reproducibility	Radiation; time; availability; resolution	Inflammation

## Data Availability

Not applicable.
